# Skull–meninges–brain connectivity and extra-axial brain tumours

**DOI:** 10.1093/braincomms/fcaf311

**Published:** 2025-08-21

**Authors:** Abdurrahman I Islim, Alexandros Vyziotis, Omar N Pathmanaban, David J Coope, Andrew T King, David Brough, Laura Jardine, Kevin N Couper, Andrew D Greenhalgh

**Affiliations:** Geoffrey Jefferson Brain Research Centre, Manchester Academic Health Science Centre, Northern Care Alliance NHS Foundation Trust, University of Manchester, Manchester M6 8FJ, UK; Division of Immunology, Immunity to Infection and Respiratory Medicine, School of Biological Sciences, Faculty of Biology, Medicine and Health, University of Manchester, Manchester M13 9PT, UK; Geoffrey Jefferson Brain Research Centre, Manchester Academic Health Science Centre, Northern Care Alliance NHS Foundation Trust, University of Manchester, Manchester M6 8FJ, UK; Geoffrey Jefferson Brain Research Centre, Manchester Academic Health Science Centre, Northern Care Alliance NHS Foundation Trust, University of Manchester, Manchester M6 8FJ, UK; Division of Neuroscience and Experimental Psychology, School of Biological Sciences, Faculty of Biology, Medicine and Health, University of Manchester, Manchester M13 9PT, UK; Geoffrey Jefferson Brain Research Centre, Manchester Academic Health Science Centre, Northern Care Alliance NHS Foundation Trust, University of Manchester, Manchester M6 8FJ, UK; Division of Cancer Sciences, School of Medical Sciences, Faculty of Biology, Medicine and Health, University of Manchester, Manchester M13 9PT, UK; Geoffrey Jefferson Brain Research Centre, Manchester Academic Health Science Centre, Northern Care Alliance NHS Foundation Trust, University of Manchester, Manchester M6 8FJ, UK; Division of Neuroscience and Experimental Psychology, School of Biological Sciences, Faculty of Biology, Medicine and Health, University of Manchester, Manchester M13 9PT, UK; Geoffrey Jefferson Brain Research Centre, Manchester Academic Health Science Centre, Northern Care Alliance NHS Foundation Trust, University of Manchester, Manchester M6 8FJ, UK; Division of Neuroscience and Experimental Psychology, School of Biological Sciences, Faculty of Biology, Medicine and Health, University of Manchester, Manchester M13 9PT, UK; Biosciences Institute, Newcastle University, Newcastle upon Tyne NE2 4HH, UK; Northern Centre for Cancer Care, Freeman Hospital, Newcastle upon Tyne Hospitals NHS Foundation Trust, Newcastle upon Tyne NE7 7DN, UK; Geoffrey Jefferson Brain Research Centre, Manchester Academic Health Science Centre, Northern Care Alliance NHS Foundation Trust, University of Manchester, Manchester M6 8FJ, UK; Division of Immunology, Immunity to Infection and Respiratory Medicine, School of Biological Sciences, Faculty of Biology, Medicine and Health, University of Manchester, Manchester M13 9PT, UK; Geoffrey Jefferson Brain Research Centre, Manchester Academic Health Science Centre, Northern Care Alliance NHS Foundation Trust, University of Manchester, Manchester M6 8FJ, UK; Division of Immunology, Immunity to Infection and Respiratory Medicine, School of Biological Sciences, Faculty of Biology, Medicine and Health, University of Manchester, Manchester M13 9PT, UK

**Keywords:** meninges, immune, lymphatics, meningioma, vestibular schwannoma

## Abstract

The cortex of the brain is covered by three meningeal layers: the dura, the arachnoid, and the pia mater. Substantial discoveries have been made demonstrating the structural and functional relationships between these layers, and with other neighbouring structures such as the skull. Importantly, improved understanding of the meningeal lymphatic network places the meninges at the nexus of a cross talk between the brain, peripheral immune system, and the skull bone marrow. The meningeal lymphatic network has been shown to regulate immune responses in models of health and disease states, such as intra-axial brain tumours, affecting a tumour’s behaviour. Unsurprisingly, a diverse array of resident and circulating immune cells such as macrophages, T-cells and B-cells can be found in the meninges, with specialized organizations or hubs surrounding the dural venous sinuses and cranial nerves. Meningioma and vestibular schwannoma are the most common extra-axial brain tumours, with varying clinical courses related to their immune microenvironments. These tumours commonly occur in proximity to the immune hubs of the meninges. This could point towards a possible bidirectional interaction, not only implicated in regulating tumour immune cell infiltration, but also meningeal inflammation and symptoms such as headaches and anxiety. This review will summarize the meningeal structure and function and highlight how these may be linked to patients with meningioma or vestibular schwannoma.

## Teaser

Islim *et al*. provide an overview of meningeal anatomy and function, illustrating how the skull–meninges–brain immunological axis can both orchestrate the behaviour of extra-axial brain tumours—such as meningiomas and vestibular schwannomas—and drive tumour symptoms like headache.

## Graphical Abstract

**Graphical Abstract:**
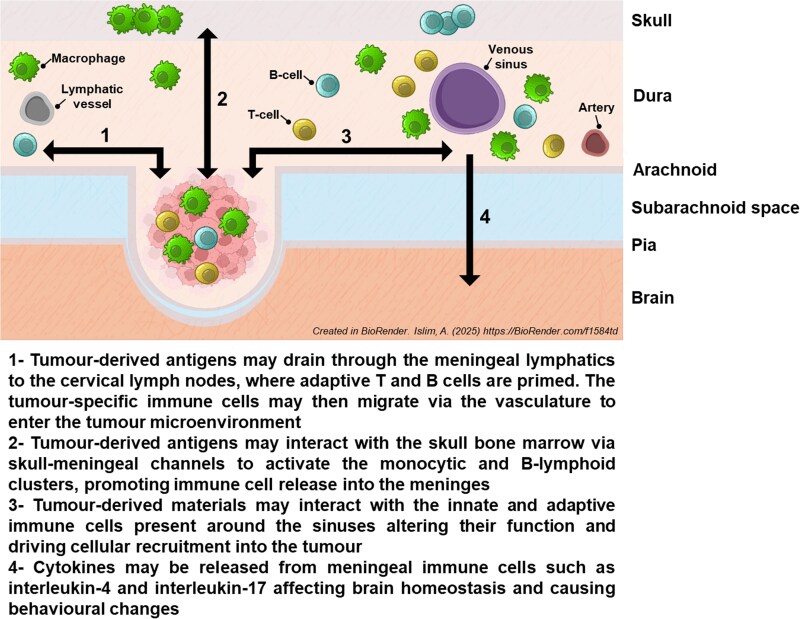

## The anatomy and structure of the meninges

### Dura mater

The dura mater in humans is a thick dense fibrous membrane. It varies in its adherence to the skull, based on age and location. For example, it is less adherent in children and at the convexity, but becomes more adherent with older age and at the skull base.^1^ The dura forms extensions to encompass the venous sinuses, and to compartmentalize the brain. For example, the falx cerebri separates the right and left hemispheres of the cerebrum and the tentorium cerebelli separates the cerebrum from the cerebellum.^2^

The dura is highly vascular. It derives its blood supply from branches of the internal carotid and external carotid arteries. Of those branches, most blood supply is attributed to the middle meningeal artery, which supplies most of the dura of the cranial convexity.^3^ The frontobasal dura and the occipito-medial dura are supplied by the anterior meningeal and posterior meningeal arteries, arising from the ophthalmic and occipital arteries, respectively.^4^ The blood supply of the dura of the skull base is likewise mostly attributed to the middle meningeal artery, and also branches of the occipital artery and ascending pharyngeal artery.^3,4^ Venous drainage of the dura is via bridging veins, into the dural venous sinuses, which in turn drain into the internal jugular vein.^5^ The blood vessels in the dura mater are fenestrated making them permeable to plasma components and circulating cells.^6^

In addition to blood vessels, corresponding with the above arteries and dural venous sinuses, the dura also consists of multiple layers of fibroblasts, sensory and autonomic nerves and lymphatic vessels, embedded within a collagen-rich extracellular matrix.^7^ Dural fibroblasts, in mouse and human, are molecularly distinguished from arachnoid and pia fibroblasts; specific markers such as *MGP* and *CRABP2* enable accurate delineation from other meningeal layers.^8,9^ Additionally, dural fibroblasts participate in functional pathways such as extracellular matrix organization, via expression of *Col4a1, Col4a2 and Col12a1* (type four and 12 collagen), major isoforms of collagen in the meninges,^10^ and immune cell regulation, discussed later.^9^ Sensory nerves of the dura are afferent branches of the trigeminal nerve, and specifically the ophthalmic division.^11^ Autonomic nerves may be sympathetic or parasympathetic, arising from the superior cervical ganglion, or otic and sphenopalatine ganglia, respectively.^12^ The network of dural lymphatic vessels was ‘re’discovered in 2015 and has been shown to drain brain interstitial fluid (ISF) and cerebrospinal fluid (CSF) from the subarachnoid space, into the cervical lymph nodes.^13,14^

The dura lymphatic vessels are predominantly found alongside the major dural venous sinuses such as the superior sagittal, transverse, sigmoid and cavernous sinuses, but could also be found throughout the whole dura.^15,16^ They are also found alongside the cranial nerves exiting through their foramina, as the dura mater forms the most superficial covering of the cranial nerves, the epineurium.^17-19^ The dural lymphatic vessels are organized into two networks: dorsal and basal, which are explored later.

### Arachnoid mater and subarachnoid space

The arachnoid mater (first leptomeningeal layer) is an avascular layer, which is separated from the more superficial dura mater, by the dural border cells.^20^ These cells are considered a constituent of both meningeal membranes, by virtue of some cells staying attached to the arachnoid, whilst others separate from the dura when taking the skull caps off in mice.^21,22^ The dural border cells are flattened fibroblasts with little to no adherens or tight junctions between them, and with no intervening collagen fibres.

In mouse and human studies, the arachnoid mater is formed by 3–4 distinct molecular groups of fibroblasts, arranged into an outer arachnoid layer, also known as the arachnoid barrier cells layer, and the inner arachnoid layer.^7,22,23^ The cells of the outer arachnoid layer adhere tightly to each other, and to the inner surface of the dural border cells, with claudin-11 tight junctions and E-cadherin adherens junctions.^22,24,25^ The presence of these junctions limits the cellular movement across the continuous outer arachnoid layer. This in addition to the presence of transporters and pumps in its cellular membrane, allows it to act as a barrier between the dura (and its contents) and the cerebrospinal fluid (blood-CSF barrier).^26^ The inner arachnoid layer is formed of two distinct fibroblast types, which are not as tightly packed as the arachnoid barrier cells, and are connected by collagen containing gap junctions and by VE-cadherin junctions.^27^

Deeper to the inner arachnoid layer is the subarachnoid space. Traversing the CSF-filled subarachnoid space are trabeculae, made of collagen, and lined by fibroblasts, like those of the inner arachnoid layer.^27^ These trabeculae can be tree shaped or rod-shaped connecting the inner layer of the arachnoid to the pia, providing a stabilizing scaffold.^28^ The distribution of arachnoid trabeculae across the brain varies, with a higher density observed in the superior regions of the brain and surrounding large blood vessels in fissures and cisterns of the subarachnoid space.^29^

### Pia Mater and subpial space

Unlike the dura and the arachnoid, which do not follow the brain parenchyma along its entire sulcal surface, the pia (second leptomeningeal layer) is a single thin layer which does. Made of flattened, transcriptionally distinct fibroblasts, pial cells are joined by VE-cadherin junctions but not tight junctions.^7,22,27^ The pia also has scattered stomas, which together with the absence of tight junctions, enable the passage of macromolecules from the subarachnoid space into the subpial space and vice versa.^30^ The subpial space contains a network of collagen fibres, which separate it from the brain parenchyma, and specifically the most superficial layer, the glia limitans. Forming part of the blood–brain barrier, the glia limitans is a thin layer composed of astrocyte processes, which surrounds the surface of the whole brain.^31^

### The relationship between the meninges and neighbouring structures such as the skull and dural venous sinuses

**Figure 1:**
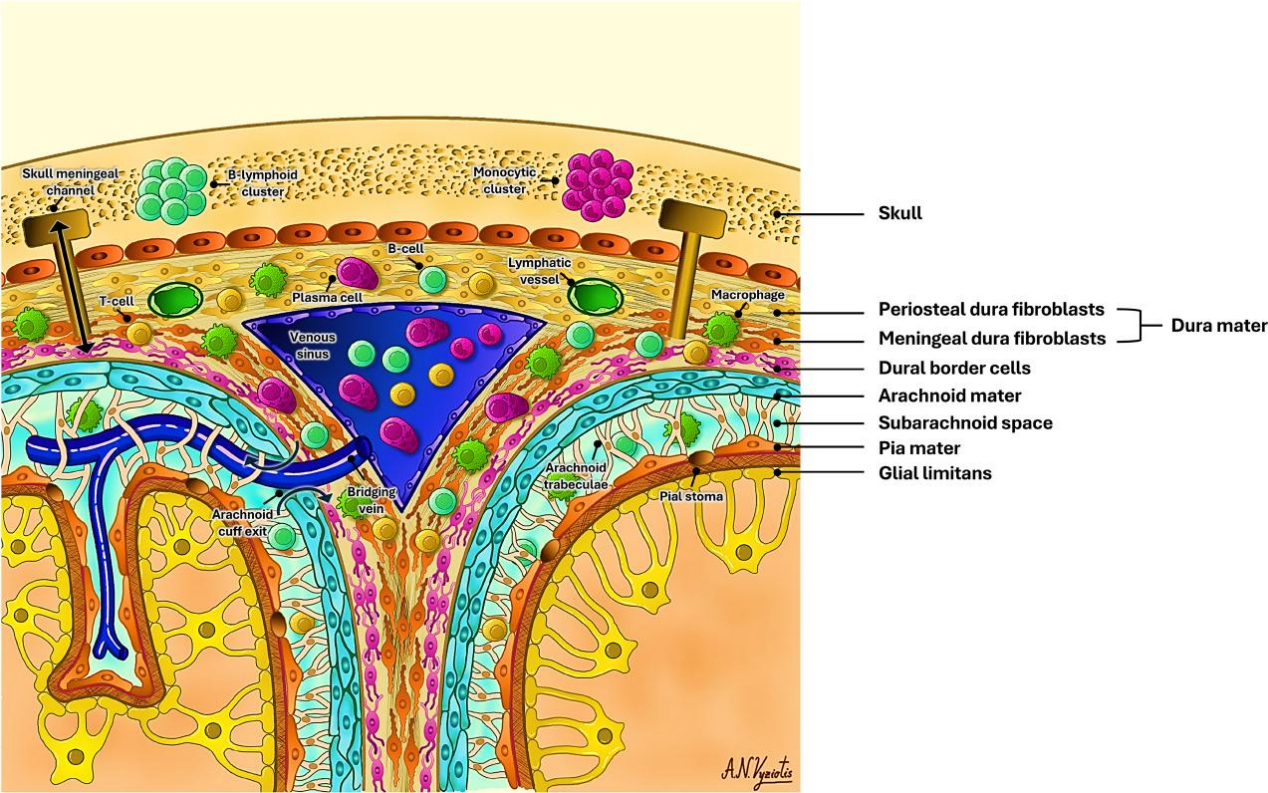
**A schematic description of the meninges and their structures**. Connecting the skull bone marrow and the dura are skull–meningeal channels, which allow bidirectional exchange of immune cells, antigens and signalling molecules. The dural venous sinuses drain the brain parenchyma venous return via bridging veins. At the point of entry of these veins into the dura are stomas which have been called the arachnoid cuff exits (ACE), which allow bidirectional exchange of CSF and its components such as immune cells and antigens, across an otherwise impermeable membrane. Surrounding dural venous sinuses are a variety of immune cells such as macrophages, T-cells, B-cells, and plasma cells. Running alongside the dural venous sinuses are the lymphatic vessels, which are thought to contribute to draining CSF, into the cervical lymph nodes.

Multiple studies have now reported the presence of channels between the skull bone marrow and the meninges, labelled as the skull–meningeal channels.^32-34^ In mouse studies, these channels have been shown to permit bidirectional trafficking of immune cells, signalling molecules and antigens between the skull and the subarachnoid space.^35,36^ Conversely, in humans, one study showed these channels most often traverse the dura, to open into the subdural space, and not the subarachnoid space.^32^ Cerebral veins and venules, typically drain into the closest dural venous sinus, via bridging veins. A recent investigation showed these bridging veins are surrounded by arachnoid barrier cells in the subarachnoid space but lose this cuff as the vein enters the dura. The loss of this cuff around the vein creates a space or a stoma in the arachnoid barrier cell layer, which has been shown to allow bidirectional exchange of molecules between the CSF and the dura. These stomas have been labelled as arachnoid cuff exits (ACEs),^37^ and may, in addition to the skull–meningeal channels, explain the skull–meninges–brain communication. Although there is non-invasive, magnetic resonance imaging (MRI) evidence pointing to the existence of ACEs in humans, these are still to be characterized in human meninges (Fig. 1).^37^

The dural venous sinuses have also been structures which attracted attention in terms of their functional relationship with the meninges. Granulations projecting from the arachnoid into these sinuses have long been considered primary sites of CSF exit in humans, down a one-way pressure gradient.^38,39^ However, a recent MRI study of 120 humans with no CSF pathologies, showed 85% of individuals at age 2 years, 15% at age 60 years and 35% at age 80 years, had no arachnoid granulations in the major dural venous sinuses.^40^ Additionally, other species such as mice do not have arachnoid granulations.^41^ Nonetheless, a recent investigation of these structures showed their capsule was an extension of the arachnoid mater, but with occasional gaps or crypts. The core of these granulations was rich with collagen, arranged in fibrillar and trabecular pattern, and often covered with arachnoid cells, which is consistent with it being an extension of the arachnoid trabeculae. In mouse, outflow of CSF and ISF, is thought to occur through the network of lymphatic vessels present along the venous sinuses, cranial nerves, and nasal plexus, and not through arachnoid granulations.^15,42^ In human, there is no clear evidence on the proportion of CSF and ISF uptake by each route.^43^

## The meningeal lymphatic network: from animal models to human

Since the re‘discovery’ of the meningeal lymphatic vessels,^13^ much effort has gone into describing the structure and function of these in mouse and in human. Recent studies have focused on the distinct roles played by the dorsal network, centred around the superior sagittal and transverse sinuses, and the basal network, placed along the skull base and its foramina.^44,45^ In mouse studies, tracers injected into the CSF, and immune cells, such as T cells, could be traced along the dorsal lymphatic vessels to the cervical lymph nodes. Additionally, ablation of these vessels seemed to alter the clinical trajectory of parenchymal diseases such as multiple sclerosis (improved via reduced trafficking of T-cells) and Alzheimer’s disease (worsened via increased accumulation of β-amyloid proteins), demonstrating how dorsal lymphatic vessels may play a part in brain ISF efflux and regulating diseases of the central nervous system.^46,47^ A later mouse study, using non-invasive imaging techniques, showed CSF drainage followed primarily the cranial nerves as they exit the foramina, with no apparent tracer uptake by the dorsal vessels.^41^ Building up on that, mouse basal lymphatic vessels have been shown in a study to have characteristics resembling collecting lymphatic vessels of peripheral organs, such as the presence of valves and button-like junctions, unlike dorsal vessels.^44^ Consistent with the previous study,^41^ the authors also highlighted the presence of tracer uptake alongside basal vessels but not dorsal vessels.^44^ More recently, a study used live imaging of intact mice skull to demonstrate tracers injected into the spinal or cranial CSF were taken up preferentially by dural lymphatic vessels, specifically those alongside the cavernous sinus.^15^ Moreover, these lymphatic vessels formed a network, which ultimately drained the sampled tracer into the deep, mandibular and accessory mandibular cervical lymph nodes, through lymphatic vessels exiting through the skull base foramina.^15^ An additional route proposed for CSF drainage, via the cribriform plate, is the nasal plexus,^45^ which may become activated under disease conditions to aid with lymphatic drainage.^42,48^ However, one study could not find a direct anatomical connection between the nasal mucosa and the meningeal lymphatic vessels, despite the detection of CSF tracers in the former.^15^

Charting of the meningeal lymphatic networks in human has been possible through non-invasive MRI. In a study of 81 patients with epilepsy, non-contrast enhanced MRI identified dorsal and basal lymphatic vessels. Outflow of CSF-ISF was detected in both dorsal and basal networks, albeit the signal across basal structures was stronger.^18^ Likewise, a study of 11 patients with various neurological disorders (e.g. idiopathic intracranial hypertension and multiple sclerosis) detected slow flow circuits of contrast around all dural venous sinuses, indicative of the presence of dural vessels. Additionally, in agreement with previous observations, the intensity of the signal around the cavernous sinus was higher. Interestingly, slow-flow channels connecting the skull and the dorsal dura could be observed, supportive of the notion of the skull–meningeal channels.^15^ With regards to the role played by the nasal mucosa, two MRI studies failed to demonstrate evidence of lymphatic drainage into the nasal mucosa through the cribriform plate,^15,49^ although a post-mortem study was able to visualize a CSF tracer injected into the cisterna magna in the nasal mucosa.^50^

**Figure 2:**
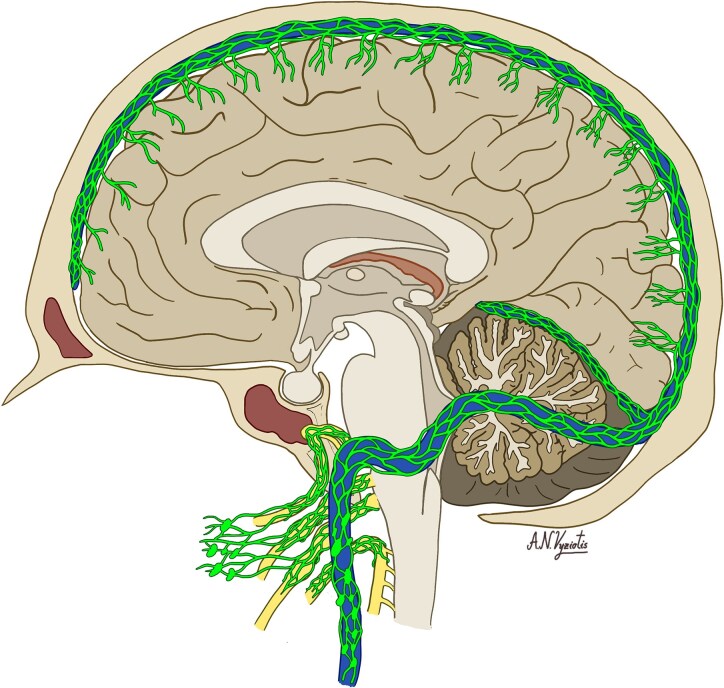
**The meningeal lymphatic network**. Lymphatic vessels run in the dura alongside the superior sagittal sinus, transverse sinus, confluence of sinuses, and sigmoid sinus, and alongside cranial nerves before draining into the cervical lymph nodes.

In summary, it is now widely accepted that the dorsal and basal lymphatic vessels (Fig. 2) contribute to draining brain ISF and CSF, linking the lymphatic system of the brain, to the peripheral immune organs. These place the dura and the other meningeal membranes at the centre of a cross talk between the brain, peripheral immune system and the skull.

## Immune cells in the meninges

Unsurprisingly, a wide and rich variety of myeloid and lymphoid immune cells have been described in mouse and human meninges, enabling innate and adaptive immunity. Myeloid cells are those most commonly found, and specifically macrophages.^9^

Based predominantly on mouse data, meningeal macrophages, also known as border-associated macrophages, are thought to originate from the yolk sac during embryogenesis. Like other tissue-resident macrophages, these are thought to have self-renewal properties.^51,52^ However, it’s also been demonstrated that dural macrophages, more than leptomeningeal macrophages, could be variably replenished by circulating peripheral and skull bone marrow derived monocytes, owing to their direct communication with the vasculature and skull.^36,53-55^ The location of these macrophages also seems to dictate their transcriptional and functional profile.^51,54^ Border-associated macrophages express varying levels of identifying molecules such as major histocompatibility complex II (MHCII), lymphatic vessel endothelial hyaluronan receptor (Lyve1), haemoglobin–haptoglobin scavenger receptor (CD163) and mannose receptor CD206 (59). The dura mater harbours a larger proportion of MHCII^+^ macrophages, compared to other layers. A few dural MHCII^+^ macrophages express C-C chemokine receptor type 2 (CCR2), which may be indicative of a monocytic origin.^56^ More single positive MHCII^+^ macrophages have been observed around ACE points in adult mice, where venules leave the subarachnoid space to join the dural venous sinuses.^57^ These macrophages have been found to interact with cells of the vessel wall (mural and endothelial cell) and fibroblasts to regulate trafficking of T-cells in response to antigens.^23,58,59^ Leptomeningeal and perivascular macrophages seem to be more commonly MHCII^-^, although MHCII^+,^ Lyve1^+^ macrophages have also been observed. Overall, border-associated macrophages are heterogeneous, with functions ascribed ranging from phagocytosis, antigen presentation and immune cell activation/recruitment (MHCII^+^ and CCR2^+^) to extracellular matrix organization and angiogenesis (Lyve1^+^).^56,60,61^

T-cells and B-cells are other common types of immune cells found in the meninges. In a healthy state, these are enriched in the dura and around the sinuses, with fewer amounts found in the leptomeninges.^62^ Studies of mouse and human dura described the presence of a wide range of T-cells, including naïve and antigen experienced subtypes.^9,63^ These included CD4^+^ effector memory, CD8^+^ cytotoxic and CD8^+^ effector memory T-cells, with CD4^+^ being most prevalent.^9,64^ Activated T-cells, in the peripheral circulation, survey the dura and respond to cognate antigens presented by the various MHCII^+^ border-associated macrophages, to play a role in immunity and autoimmunity.^9,57,65^ Gamma-delta (γδ) T-cells have also been observed in both mouse and human dura and are thought to conversely have a tissue-resident phenotype, with ties to behaviour and short memory, via interleukin 17a (IL-17a) mediated signalling of neurons.^64,66^ Other T-cell derived cytokines such as interferon-gamma (IFN-γ) and IL-4 have also been linked with behaviour and memory.^63,67^ B-cells have also been observed in mouse and human meninges, and particularly in the dura close to venous sinuses.^9,23,68^ The majority of these cells are thought to have a tissue-resident phenotype, replenishable by the skull bone marrow, with cells existing on a spectrum from pro-B cells to mature, immunologically tolerant, B-cells.^68,69^ This maturation process appears to occur in the dura itself, dependent on C-X-C Motif Chemokine Ligand 12 (CXCL12), produced by fibroblasts.^69^ On the other hand, other specialized B-cell populations appear to arise from peripheral tissues. For example, aged B cells infiltrate the dura of aged mice from systemic circulation, with a possible role in autoimmunity by sustained production of autoantibodies, upon activation by local antigens.^69^ IgA-secreting plasma cells have been shown to be recruited form the mouse gut to the meninges, residing primarily around the sinuses.^70^ Recently, organized germinal-centre-like aggregates of CD4^+^ T-cells, B-cells and plasma cells, around the dorsal and ventral meningeal networks, have been described in mouse and human and annotated as dural-associated lymphoid tissue (DALT).^71^ These observations indicate the dura may be a site for antigen sampling, where humoral immune responses can take place.^72^

Overall, the meninges, and specifically the dura, are populated with a diverse array of resident and patrolling immune cells, with roles in innate and adaptive immunity.

## Examples of the influence of the meningeal immune landscape on brain tumours

Evidence has started to emerge on the role of the meningeal immunity in regulation of intrinsic brain tumours, such as medulloblastoma and glioma.^73-76^ Medulloblastoma is the most common malignant, WHO grade 4, tumour in children and arises in the cerebellum.^77^ Molecularly, these tumours can be divided into four subgroups: Wingless (WNT, group 1), sonic hedgehog (SHH, group 2), group 3 and group 4. WNT and SHH medulloblastomas are primarily driven by mutations in signalling pathways (Wingless and Sonic hedgehog) while the other two have a less clear molecular basis.^78^ In mouse models of SHH medulloblastoma, Lyve1^+^ meningeal macrophages were found to have a tumour suppressive function via secretion of CXCL4, and their depletion led to tumour growth.^73^ Glioblastoma (GBM) is the most common malignant brain tumour in adults.^79^ The tumour microenvironment of a GBM is complex and characterized by marked inter- and intra-tumoural heterogeneity.^80^ Nonetheless, macrophages, T-cells and their interactions, within different niches of the tumour, appear to have an effect on the tumour’s response to treatment.^81-83^ In two studies of GBM, involving mouse models and human tissue, the skull bone marrow was found to contribute antigen presenting myeloid cells and lymphoid cells to the tumour microenvironment.^75,76^ Furthermore, increased activation of these cells within the skull bone marrow, detectable via MRI, and induced using a stem cell mobilizer, improved tumour control. Lymphoid cell infiltration into a GBM also appears to be regulated by the drainage efficiency of the meningeal lymphatic networks; intrathecal treatment with VEGF-C increased CD8^+^ T-cell entry and improved responsiveness to immune checkpoint blockade.^74^ What remains unclear are the signalling mechanisms behind trafficking of these immune cells, and how the various meningeal layers, and their constituents, regulate this process.

## What evidence is there to the relationship between the meninges and extra-axial brain tumours?

### The clinical behaviour of meningioma and vestibular schwannoma is dictated by their rich immune landscape

#### Meningioma

Meningioma are the most common primary brain tumours (40%), with an incidence rate of 9.7/100 000.^79^ There are 15 histopathological subtypes, classed as either WHO grade 1,2 or 3, with approximately 80% being non-malignant WHO grade 1. The remainder are grade 2 or 3, with a higher tendency to recur.^84^ These tumours are mostly sporadic, however patients who had previous exposure to cranial radiation (radiation-induced meningioma), and patients with *NF2*-schwannomatosis (*NF2*-SWN) have a tendency to develop more aggressive and multiple tumours.^85,86^ Sporadic tumours have a variety of recurrent oncogenic mutations; most are *NF2*-mutated, but others, which are mutually exclusive with *NF2*, include *TRAF7, AKT1, KLF4, SMO* and *PIK3CA.*^87^

Active monitoring is recommended for the management of a patient with an asymptomatic meningioma, to monitor for growth and/or clinical progression.^88^ Surgery is the first line management option for a symptomatic or a growing meningioma. Most meningioma are ‘cured’ by surgical resection alone, however, a small proportion of patients experience recurrence, necessitating repeat surgery or radiotherapy. Despite these conventional salvage treatment methods, a minority of patients may experience complete loss of disease control, with no other effective therapies to offer.^89,90^ The immune tumour microenvironment of a meningioma primarily comprises of macrophages, T-cells and B-cells. These have all been shown to exert an effect on the clinical behaviour of a meningioma.

In an immunofluorescence study of 30 patients (16 grade 1, 14 grades 2 and 3), tumour-associated macrophages accounted for 18% of all cells and were predominantly of a pro-tumoural, anti-inflammatory (alternatively activated) subtype.^91^ Additionally, it was found that an increased proportion of pro-tumoural to anti-tumoural macrophages (classically activated) increased the risk of meningioma recurrence after surgery. Another study found an increased expression of colony stimulating factor 1 (CSF1) in the plasma of patients with grades 2 and 3 meningioma, thought to be responsible for polarizing macrophages towards an alternatively activated phenotype.^92^ An increased infiltration of CD4^+^ helper, and CD8^+^ cytotoxic T-cells appears to reduce the risk of meningioma recurrence,^93^ however CD8^+^ regulatory T-cells seem to be associated with a higher meningioma grade and an increased risk of recurrence.^94^ A subset of T-cells in meningioma have an exhaustive phenotype, characterizable by the presence of the immune checkpoint molecule programmed-death 1 (PD-1)^95^; this has also been found to increase the risk of meningioma recurrence.^96^ B-cells are rarer in meningioma but seem to have an antigen experienced phenotype, suggestive of maturation elsewhere in a germinal centre (e.g. DALT).^95^ A study of 93 patients with a meningioma found a higher expression level of CD20^+^ B-cells in tumours which recurred versus those which did not.^97^

Meningioma appear to also differ in their immunological status based on their location and aetiology. Skull-base meningioma, which tend to have a lower recurrence and growth rates than convexity meningioma,^98^ were found in a network analysis to be enriched for γδ T-cells.^99^ Recently, a molecular classification of sporadic meningioma divided them into four molecular groups (MG1-4). MG1, the most indolent group, were enriched for proteins involved in immunoregulation, and characterized by *NF2* mutations.^100^  *NF2*-SWN associated meningioma appear likewise to have a marked immune cell infiltration, primarily with macrophages.^101^

#### Vestibular schwannoma

VSs are the most common non-malignant nerve sheath tumours, with an incidence rate of 1.50 per 100 000 population.^79^ VSs are WHO grade 1 and mostly sporadic. However, they also form the hallmark feature of *NF2*-SWN, when found bilaterally, and may arise unilaterally in 5% of patients with *LZTR1*-related schwannomatosis.^102,103^ Most sporadic VSs harbour somatic mutations in *NF2* or genes linked to it.^104,105^ Unlike meningioma, VS is recognized as one histopathological entity but with dense (Antoni A) and loose cellular (Antoni B) areas.

Active monitoring is recommended for patients with asymptomatic or minimally symptomatic VSs, to monitor for growth and clinical progression. Surgery or radiotherapy may be recommended for patients with a growing or a symptomatic VS.^106^ In patients with *NF2*-SWN, off-label treatment with bevacizumab (Avastin®), a humanized antibody that inhibits vascular endothelial growth factor (VEGF), is indicated for a growing or symptomatic VS.^107^

A VS and its treatment may have considerable effects on a patient’s quality of life. Persistent growth of a VS leads to unilateral hearing loss, which in the context of *NF2*-SWN and bilateral VSs, could lead to deafness. Surgical treatment of a VS is not without risks; 10–17% of patients have a facial nerve deficit at last follow-up and up to 30% of patients experience complications such as CSF leak, infection, haematoma, or hydrocephalus, requiring a repeat operation.^108,109^ Consideration must be given to this in patients with *NF2*-SWN, given the high number of surgical procedures patients are likely to undergo in their lifetimes, for VSs and other tumours such as meningioma and ependymoma.^110^ Recurrence after a subtotal resection, which may be intended to reduce the risk of complications (∼30% of patients), arises in about a third of patients necessitating salvage surgery or radiotherapy. Radiotherapy, including stereotactic radiosurgery, carries risks such as facial nerve deficit (10%), and specifically for patients with *NF2*-SWN,^111^ an increase by 5% to the incidence rate of a malignant tumour.^112^ Bevacizumab side effects include fatigue (34%), hypertension (30%) and bleeding (e.g. epistaxis [10%] and menorrhagia [28%]) and unfortunately some patients with *NF2*-SWN will become refractory to treatment or demonstrate rapid growth of their VS after cessation of treatment due to side effects.^113,114^ In view of the aforementioned factors, better treatments for sporadic and NF2-SWN VS have been sought. By studying the tumour microenvironment of a VS, studies have shown that it is highly composed of immune cells, particularly macrophages and T-cells, augmenting the tumour’s behaviour.^115^

Early histopathology studies showed an increased density of macrophages in growing and recurrent *versus* static VSs, albeit the phenotype was inconsistent; pro-tumoural, alternatively activated in one study and anti-tumoural, classically activated in another.^116^ More recently, single cell transcriptomic studies demonstrated VS macrophages take on a range of states, but could be broadly fitted into classically activated, alternatively activated and transitioning monocyte classes.^117,118^ A spatial protein-targeted study demonstrated an increased abundance of alternatively activated macrophages, compared to classically activated macrophages, in both growing and static VS, but that the relationship with other cells differed; classically activated macrophages colocalized and inhibited alternatively activated macrophages in static tumours, but alternatively activated macrophages were sequestered from this check by CD8^+^ T-cells and transitioning monocytes in growing tumours.^119^ Leading on from this, a recent transcriptomic study showed recurrent VSs were enriched with senescent CD4+ and CD8+ T-cells.^120^ An exhaustive phenotype of T-cells, characterized by PD-1, has been shown in one histological study to correlate with tumour growth but single-cell RNA studies have been inconsistent.^121,122^ Furthermore, a high dimensional imaging study demonstrated CD8^+^ T-cells were blocked from tumour cells by other T-cells and classically activated macrophages in Antoni A, compared to Antoni B regions, but the significance of this with relation to tumour growth is yet to be determined.^123^ Interestingly, the immune landscapes of sporadic and *NF2*-SWN VS seem to be similar, based on histological, proteomic and transcriptomic data.^124,125^

### Meningioma and vestibular schwannomas are anatomically related to the immune hubs of the meninges

Meningioma are thought to arise from either the dural border cells, or the arachnoid barrier cells of the meninges.^126,127^ VSs arise from the Schwann cells of the vestibulocochlear nerve (8th cranial nerve), with its epineurium considered an extension of the dura. Meningioma commonly have a relationship with a neighbouring dural venous sinus (superior sagittal, sigmoid, transverse or cavernous). VSs, by virtue of their location within the cerebellopontine angle, are in close proximity to the sigmoid sinus.^128^ These factors would indicate that these tumours are first, highly amenable to infiltration and regulation by the wide repertoire of immune cells, present in the dura close to the venous sinuses, and second, able to influence the dura and its contents, such as the lymphatic vessels and nerves. In support, a single-cell study pointed towards shared macrophage, T-cell and B-cell clones between paired dura, distant from tumour, and meningioma samples.^9^ Such studies are yet to take place for VS, albeit a single-cell study concluded infiltrating macrophages were unlikely to be of a microglial origin, but rather bone-marrow derived.^117^ For meningioma arising from the dorsal dura, alongside the superior sagittal sinus, there is also the possibility of an interaction with the skull bone marrow and its immune cells, via skull–meningeal channels.^15^ This may be of relevance for skull-base meningioma and VS, however no studies to date have observed skull-base meningeal channels. This is most likely due to the difficulty in sampling skull-base dura in mouse and human, and imaging them at a high resolution. To affect brain function, e.g. memory and behaviour, meningeal cytokines such as IL-17, IL-4 and IFN-γ need to be able to access the subarachnoid space, where CSF-ISF exchange takes place. ACEs may explain this and MRI evidence pointing towards their existence in human has started to emerge.^37^ Additionally, both meningioma and VSs partly interface with the subarachnoid space, altering the proteomic landscape of CSF.^129-132^

Overall, both meningioma and VS arise in proximity to the immune hubs of the meninges, where they may be influenced by meningeal immune cells, their interactions, and produced cytokines affecting brain function.

### Unexplained patient symptoms and clinical findings, and their link to immune cell response in the meninges

Meningioma typically present with seizures, focal neurological deficits but a large proportion may also be asymptomatic, picked up incidentally.^133^ VSs commonly present with reduced hearing and tinnitus and uncommonly with facial weakness. They also may be picked up incidentally while asymptomatic.^134^ Twenty-to-70% of patients with a VS or a meningioma present with headaches, which may not be thought to be related to the tumour.^135,136^ This is common in the context of small tumours, with no mass effect to the underlying brain, and in the absence of hydrocephalus. Likewise, conservatively managed patients, with a VS or a meningioma, may display cognitive deficits and increased anxious behaviour, in the absence of a compressive pathology and despite reassuring radiological behaviour on follow-up scans.^137-140^A biological phenomenon, underlying these symptoms and clinical findings, has begun to be explored; for example, imaging studies in vestibular schwannoma demonstrated inflammatory signals within normal appearing brain regions,^141^ and this has been linked to cognitive deficits in patients.^142^ The mechanisms underlying this, however, are not well understood but studies in other fields may shed light onto them. For example, mouse studies demonstrated migraine triggers, such as stress, increase the expression of pro-inflammatory dural macrophages, activating the afferent branches of the trigeminal nerve.^143^ Moreover, this activation appears to drive recruitment of further immune cells, such as CCR2^+^ macrophages and CD4^+^ effector memory T-cells, sustaining the sensitization of dural afferent neurons.^144^ Furthermore, reduced lymphatic clearance of immune cells appears to attenuate pain behaviour.^145^ With regards to anxiety and memory (brain function), meningeal cytokines, such as IL-17, IL-4 and IFN-γ, may be able to access the subarachnoid space via ACEs and eventually lead to neuronal activation.^64,66^ The above observations should be validated with mouse models of brain tumours, and assessment of human meningeal tissue of patients with brain tumours, taken at the time of surgery. Additionally, findings in human should be correlated with preoperative imaging findings, expanded to detect inflammation with novel MRI contrast agents (e.g. ultrasmall superparamagnetic iron oxide nanoparticles, which is taken up by macrophages) or to include other imaging modalities of inflammation such as positron emission tomography.^146,147^

## Conclusion

Recent advancements have enabled further understanding of meningeal biology, including its immune landscape and lymphatic network. The clinical behaviour of meningioma and VSs, the most common extra-axial brain tumours, are dictated by their immune status. By virtue of their locations relative to the meninges, it is highly likely a relationship between these tumours and meningeal immunity may be responsible for regulation of immune cell entry, and for symptoms and signs of disease which are not fully understood to date.

## Data Availability

Data sharing is not applicable to this article as no new data were created or analysed in this study.
